# Evaluating the safety of bipolar nephrostomy tract cauterization “BNTC” towards a safe tubeless percutaneous nephrolithotomy: a randomized controlled trial

**DOI:** 10.1007/s00240-024-01575-2

**Published:** 2024-07-18

**Authors:** Mohamed Omar, Tarek Ahmed Amin Ibrahim, Sultan Sultan, Mohamed El-Gharabawy, Yasser Noureldin, Saeed Bin Hamri, Khaled Sayedahmed

**Affiliations:** 1 Urology Department, Menoufia University, Shibin el Kom, Menoufia Egypt; 2 Urology Department, Hamad Medical Corporation, Doha, Qatar; 3 Urology Department, Benha University, Benha, Egypt; 4 Urology Department, Northern Ontario School of Medicine, Thunder Bay, ON Canada; 5 Urology Division, King Abdulaziz Medical City, Riyadh, Saudi Arabia; 6 Urology Department, Rhein-Maas Hospital, Würselen, Germany

**Keywords:** Tubeless, PCNL, Tract, Cauterization

## Abstract

To assess the safety and effectiveness of tubed versus tubeless percutaneous nephrolithotomy (PCNL) after tract inspection and bipolar cauterization of the significant bleeders. Patients who were scheduled for PCNL were screened for enrollment in this prospective randomized controlled trial. The patients were randomly assigned to one of two groups; Group 1 received tubeless PCNL with endoscopic inspection of the access tract using bipolar cauterization of the significant bleeders only, while Group 2 had a nephrostomy tube was inserted without tract inspection. We excluded patients with multiple tracts, stone clearance failure, and significant collecting system perforation. We recorded blood loss, hemoglobin drop after 6 h, postoperative analgesia requirements, hospital stay, and the need for angioembolization. A total of 110 patients completed the study. There were no significant differences between the two groups in in terms of demographic characteristics. Likewise, there was no significant difference in the mean decrease in hemoglobin after 6 h and the frequency of blood transfusion. However, the incidence of hematuria within the first 6 h (p = 0.008), postoperative pain scale (p = 0.0001), the rate of analgesia requirement (p = 0.0001) and prolonged hospital stay (p = 0.0001) were significantly higher in Group 2. Only 9 cases of tract screened patients (16% of group 1) required cauterization. Tubeless PCNL with tract inspection and cauterization of bleeders can provide a safer tubeless PCNL with less postoperative pain, analgesia requirement, and same-day discharge.

## Introduction

Percutaneous Nephrolithotomy (PCNL) is the first-line therapy for treating large stones exceeding two cm [1, 2]. Despite the high stone-free rates, PCNL poses a higher risk of morbidity than flexible ureteroscopy [3]. To reduce the postoperative morbidity resulting from pain and subsequent hospital stay, some clinical trials have recommended omitting the placement of a Nephrostomy tube (NT) in uncomplicated PCNL cases [4]. The Placement of an NT is considered a safety precaution to ensure a smooth postoperative course by draining the renal system and reducing postoperative bleeding [5, 6]. A survey conducted among endo-urologists showed that more than 60% still prefer to Keep an NT postoperatively [7]. Although recent studies have shown that total tubeless PCNL may lead to better transfusion rate and postoperative stay outcomes, the trials were appraised for including small sample sizes and small stone burdens [8]. Hence, the results could not be validated for complex cases [9].

To expand the indications of tubeless PCNL and safely avoid bleeding, some trials reported success with cauterization of the nephrostomy tract in reducing postoperative bleeding, pain, and hospital stay [10, 11]. However, to the best of our knowledge, the trials were conducted retrospectively, and an unbiased prospective randomized controlled trial (RCT) has yet to be carried out.

This study is the first RCT to investigate the efficacy of cauterization of significant bleeders upon inspection of the PCNL tract as a transition to a safer exit strategy of a tubeless PCNL. The approach aims to reduce morbidity caused by bleeding and improve patient’s outcomes related to postoperative pain and length of hospital stay.

## Materials and methods

### Study design, ethics, patient selection, and randomization

**Fig. 1:**
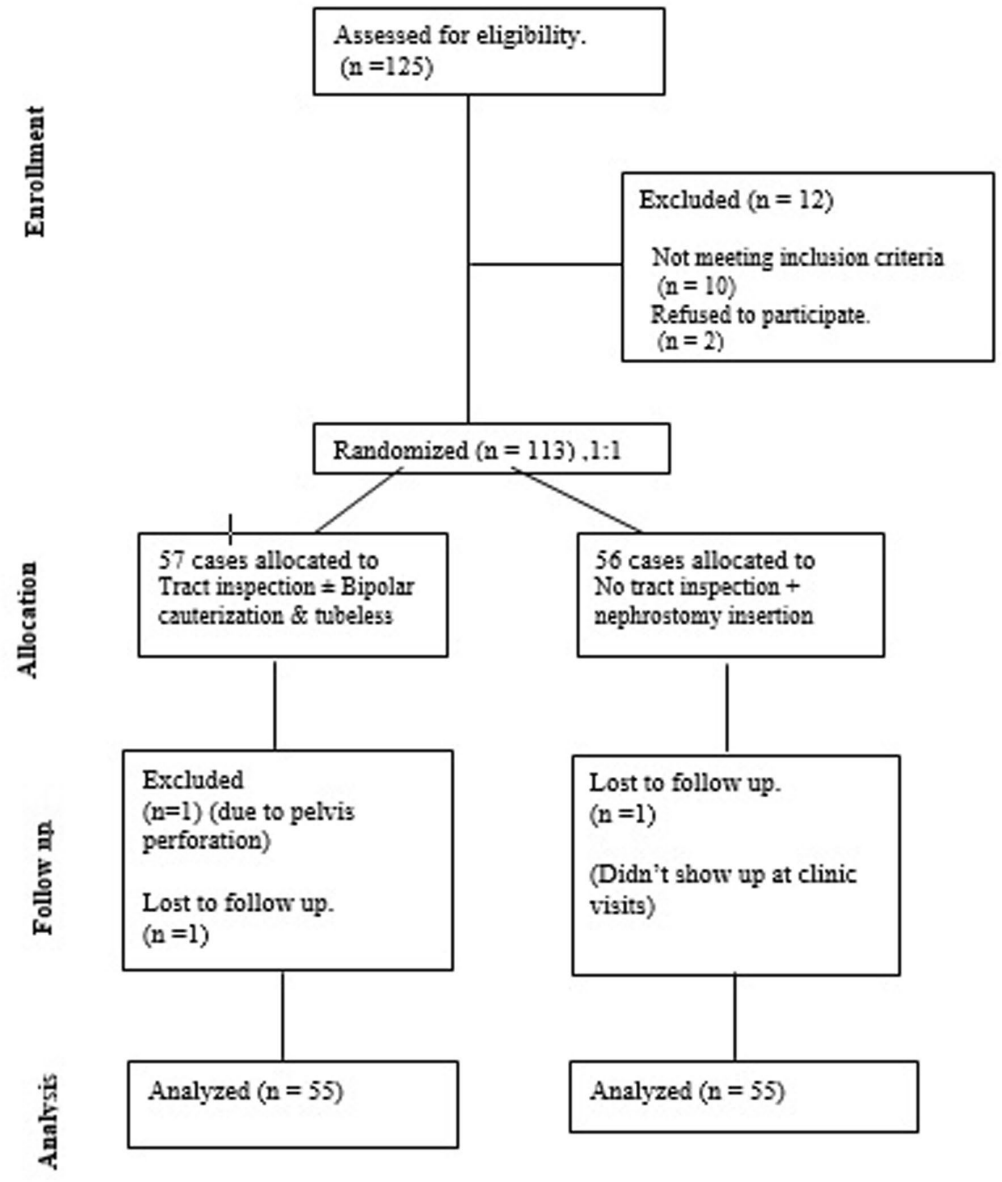
Consolidated standards of reporting trials (CONSORT) flowchart of study cases

Upon approval from the institutional review board, all patients scheduled for PCNL at Menoufia University hospitals were screened for recruitment. The study included adults over 18 years old with large renal stones exceeding 2 cm that required PCNL and stone morphology that could be accessed through a single nephrostomy tract. The exclusion criteria were patients who experienced intraoperative perforation of the pelvicalyceal system, required more than one puncture, or had significant stone residuals that required a second look PCNL. Figure 1, a CONSORT (Consolidated Standards of Reporting Trials) study diagram, summarizes the study.

The eligible patients were provided counseling and asked to sign an informed consent form. They were then randomly assigned to one of the two study groups using a computer-generated randomization table with a 1:1 allocation. Group 1 included patients who underwent tubeless PCNL after inspection of the nephrostomy tract for bleeders and bipolar cauterization (BNTC) whenever significant bleeders from renal parenchyma or muscle and subcutaneous tissue followed by tubeless exit. Group 2 underwent tubed PCNL without tract inspection or cauterization. Both groups received ante-grade Double-J ureteral stenting.

The study followed good clinical practice guidelines according to the Declaration of Helsinki. The principal investigator ensured confidentiality through coding and secure storage of patient data. Data collected included demographic information, stone characteristics, intraoperative details, post-operative stone clearance, and complications.

### Surgical procedure

A standard PCNL (30 Fr sheath) was carried out using a fluoroscopic triangulation technique while the patient was lying in a prone position after placement of a 6 Fr ureteral catheter for retrograde pyelography. The tract was then dilated using serial metal telescopic Alken dilators under fluoroscopic control. Pneumatic lithotripsy was used to fragment the stones, and the fragments were retrieved using stone forceps. Once the procedure was complete, residual stones were screened endoscopically and by fluoroscopy to ensure complete stone clearance. A Nephrostogram was performed to check for any perforations. After the procedure, to choose the exit strategy, the surgeon was endorsed the group allocation of the patient as per the pre-specified computer randomization.

In Group 1, the tract was inspected using a 26 Fr resectoscope, and any bleeding vessels at the parenchymal edge were cauterized using a bipolar ball electrode (Karl Storz Tuttlingen™, Germany). The sheath was then retracted into the muscular and subcutaneous layers, and only significant bleeders were cauterized before leaving the tract without a nephrostomy.

In Group 2, 16 Fr Nelaton catheters were inserted and kept clamped. On the first postoperative day, the catheters were de-clamped and removed after ensuring the absence of significant hematuria.

### Definitions

The surgical blood loss of a patient was determined by comparing their hemoglobin levels before and 6 h after the surgery. To ensure accurate results, no intravenous fluids were administered 2 h before blood sampling, and samples were provided to the same hospital laboratory. After the surgery, a resident assessed the patient’s need for pain relief if their pain score was higher than 6 on the standard numeric pain scale (ranging from 0 to 10). This was checked twice. Additionally, postoperative hematuria was evaluated using a visual scale designed by Stout et al. [12] For this study, grades 3, 4, or 5 were considered significant.

### Statistical analysis

A sample size calculation was conducted using the clinic calculator, which revealed that forty-seven patients per group were needed. We set the type-1 error (α) at 0.05 and power at 80%. The data from Karadeniz et al. was utilized as a reference for the anticipated difference in hemoglobin drop between both groups after 6 h as our primary endpoint [13]. The sample size was increased to accommodate excluded patients. We compared baseline patient’s characteristics between the two groups using a *t*-test for quantitative data and a chi-square test for qualitative variables. Correlations analysis (Pearson’s) was used to examine the relationship between two quantitative variables. The statistical package SPSS 20.0 was used to analyze the data, and a *p*-value of less than 0.05 was considered statistically significant.

## Results

The study’s enrollment process is visually represented by the CONSORT diagram, displayed in Fig. 1. At the beginning of the study, 125 patients were enrolled, and 15 patients dropped out, leaving 55 patients in each group. Patients who had multiple tracts, stone clearance failure, significant collecting system perforation, and patients who lost follow-ups with no show at the clinic were excluded after recruitment. Both groups had similar demographic and stone characteristics, as presented in Table 1. Table 2 illustrates the perioperative outcomes, which shows that both groups are equivalent regarding the amount of irrigation fluid used during the operation, the selected punctured calyx, and the stone-free rate (Group 1; 88% Vs. Group 2; 86%). Although the mean operative time was slightly longer for Group 1 (85 ± 38) versus Group 2 (80 ± 39), this difference was statistically insignificant (*p* = 0.3). The mean hemoglobin drop after 6 h was slightly greater in Group 2 (1 ± 0.9 g/dL) than in Group 1 (0.7 ± 0.8 g/dL), but this difference was statistically insignificant (*p* = 0.07). None of the patients in Group 1 required a blood transfusion, while two in Group 2 experienced significant bleeding postoperatively and needed a blood transfusion. However, the difference between the two groups was statistically insignificant (*p *= 0.08). Table 3 demonstrates the complications that occurred in each group according to the Clavien-Dindo classification. Group 2 had a higher incidence of hematuria in the first 6 h (*p* = 0.008), non-steroidal anti-inflammatory drug administration (*p* = 0.0001), and postoperative pain scale (*p* = 0.0001). In Group 1, nine patients (16%) required BNTC during tract inspection. Among them, one revealed arterial and venous parenchymal bleeding, three had only venous parenchymal bleeding, and four showed muscle or subcutaneous tract bleeding Table 4.

**Table 1:** Demographics and stones characteristics

Variable	Group (1): BNTC *n* = 55	Group (2): NT *n* = 55	*p*-value
Age, mean (SD)	52 (14)	54(12)	0.5
Female, *n* (%)	25 (45%)	24 (44%)	0.8
Side (Right)	24 (44%)	29 (53%)	0.3
BMI, kg/m2, mean (SD)	34 (3.5)	34.4 (3.1)	0.07
HTN, *n* (%)	22 (40%)	21 (38%)	0.8
DM, *n* (%)	17 (31%)	15 (27%)	0.6
Stone size, largest diameter, cm, mean (SD)	3.7 (1.4)	3.8 (1.1)	0.5
Stone HFU, mean (SD)	946 (348)	991 (354)	0.5

*BMI* body mass index, *HTN* hypertension, *DM* diabetes mellitus, *HFU* Hounsfield unit

**Table 2:** Peri-operative outcomes

Variable	Group (1): BNTC *n* = 55	Group (2): NT *n* = 55	*p*-value
Irrigation amount, L, mean (SD)	17.3 (8.7)	16.4 (8)	0.4
Punctured calyx,*n* (%) Upper Middle Lower	4 (7%) 10 (18%) 41 (75%)	5 (9%) 9 (16%) 41(75%)	0.8
Operative time, min, mean (SD)	85 (38)	80 (39)	0.3
Hemoglobin drop (gram/dl -6 h. post)	0.7 (0.8)	1 (0.9)	0.7
Blood transfusion	0	2 (3%)	0.9
Hematuria (1st 6 h.)	4 (7%)	14 (25%)	***0.008***
Hematuria (1st 24 h.)	2 (4%)	7 (13%)	0.07
Pos-operative analgesic administration	9 (16%)	30 (54.5%)	***0.0001***
Post-operative pain, (likert 0–10), median (range)	3 (2–4)	6 (4–7)	***0.0001***
Hospital stays, hours, mean (SD)	6.7 (5.6)	28.8 (15.5)	***0.0001***
Stone free rate (%)	86%	88%	0.8
Angio-embolization, *n* (%)	0	1(1.8%)	0.3

Bold Italics indicates statistical significance

**Table 3:** Intra and post-operative complications

Modified Clavien-Dindo grading system	Group (1): BNTC *n* = 55	Group (2): NT *n* = 55	*p*-value
Grade 1 Transient fever Analgesic requirement	4(7%) 9 (16%)	5(9%) 30(54.5%)	0.7 0.0001
Grade 2 Blood transfusion	0	2 (3%)	0.09
Grade 3a Angioembolization	0	1(1.8%)	0.3

**Table 4:** Tract inspection outcomes in Group 1

Source of bleeding		Positive bleeding *N* = 9
Renal parenchyma Bleeding (*N* = 5)	Arterial only	0
	Venous only	4
	Both	1
Extra-renal tract bleeding (*N* = 4)	Arterial only	3
	Venous only	1
	Both	0

## Discussion

Kidney stone disease is a common medical condition that is becoming more prevalent worldwide[14]. Achieving a stone-free status with minimal morbidity in a single session is crucial in managing recurring urolithiasis disease. PCNL is considered the procedure of choice for large renal stones [1, 2]. Postoperatively, the standard of care involves keeping a Nephrostomy tube to allow drainage of the collecting system and provide a safety tamponade for the access tract. While some studies have confirmed the safety of tubeless PCNL, they only included simple, uncomplicated cases and excluded complex scenarios where bleeding occurs [8, 9]. Hence, tubeless PCNL was only validated for uncomplicated cases.

As an alternative to the placement of an NT, cauterization of the tract was reported to be as efficient and safe. However, there is a lack of randomized controlled trials that provide unbiased results.

This first randomized controlled trial evaluated the haemostatic effect of BNTC in cases with significant bleeders compared to the placement of an NT. The study results revealed that BNTC is more effective in controlling post-PCNL bleeding and decreases postoperative bleeding and pain, reducing the need for analgesia and shorter hospital stays. The results are deemed reliable since the unbiased randomization process resulted in matched groups in terms of the baseline pre-operative demographic and stone characteristics. The study was conducted at a high-volume tertiary hospital in a country located within the Stone Belt, and the outcomes are comparable to those of the literature [15].

The primary outcome of the present study was to evaluate the efficiency of BNTC as a safety measure to control bleeding in cases with tubeless PCNL. Bleeding is not an uncommon complication post-PCNL, with an average rate of 7.8%, among whom 5.8% might need blood transfusion [15]. In the present study, 3% of Group 2 patients required blood transfusion, while none of Group 1 patients were transfused. Though statistically insignificant, the difference is clinically significant. Bleeding might be life-threatening, necessitating blood transfusion with the possibility of angioembolization to control arterial bleeding. Angioembolization was required for one patient in Group 2 to control arteriovenous fistula, while none of Group 1 patients needed it. It is worth mentioning that one patient in Group 1 had an arterial parenchymal edge bleeder, which was cauterized. We believe that if this artery had not been cauterized, the patient may have needed angioembolization. BNTC was not required for all the tubeless group candidates. Upon tract inspection by the end of the procedure, BNTC was done for only those with significant active bleeding noticed from the parenchymal edge or the nephrostomy tract. In Group 1, active bleeding was encountered in 9 patients (16%), while the rest did not need cauterization. A case series reported by Gupta et al. described a similar technique to cauterize only the significant bleeders upon tract inspection [16].

The present study emphasizes the advantages of tubeless PCNL as stated in the literature [8, 9]. Group 1 patients required significantly less analgesia and shorter hospital stays. In line with the present study, previous reports suggested that cauterization of the tract further reduces haematuria and pain, enhancing patient’s recovery and reducing hospital stay [10, 11]. Group 1 patients were discharged within 12 h postoperatively. The safety of same-day discharge following tubeless PCNL was validated by Chong et al. [17].

In summary, This RCT verified the superiority of BNTC with tubeless PCNL over NT; BNTC resulted in less postoperative bleeding and pain that allowed the same-day discharge of the patients. However, some limitations are to be considered; more complex cases necessitating multiple tracts were excluded to validate the safety of the relatively novel technique.

## Conclusion

Tubeless PCNL with tract inspection and cauterization of bleeders may be a safer option for generalizing tubeless PCNL, allowing same-day discharge and less postoperative pain and bleeding. Multi-institutional prospective randomized controlled trials are needed to validate the technique for complex cases and confirm its effectiveness in preventing angioembolization.

## Data Availability

Datasets are available on request.
